# Nasal microbiome and the effect of nasal decolonization with a novel povidone-iodine antiseptic solution: a prospective and randomized clinical trial

**DOI:** 10.1038/s41598-023-46792-8

**Published:** 2024-07-20

**Authors:** Diana Fernández-Rodríguez, Jeongeun Cho, Emanuele Chisari, Martin J. Citardi, Javad Parvizi

**Affiliations:** 1 Rothman Orthopaedic Institute, 125 S 9th St. Ste 1000, Philadelphia, PA 19107 USA; 2 Plan de Estudios Combinados en Medicina (PECEM), Facultad de Medicina, Universidad Nacional Autónoma de México, Mexico City, Mexico; 3 Department of Otorhinolaryngology-Head and Neck Surgery, McGovern Medical School, The University of Texas Health Science Center at Houston, Houston, TX USA

**Keywords:** Applied microbiology, Clinical microbiology

## Abstract

The aim of this study was to assess the profile of nasal microbiome and evaluate the effect of a specific nasal decolonization solution on the microbiome. We conducted a randomized, placebo-controlled, and parallel-group clinical study of 50 volunteers aged 18 years and older. The subjects were randomly assigned to receive a nasal antiseptic solution, containing povidone-iodine as the main ingredient, (n = 25) or a control solution (n = 25). Nasal swabs were obtained before application (baseline) and at 3 timepoints after application (5 min, 2 h, 24 h). Nasal swabs were subjected to next generation sequencing analysis and cultured in agar plates. At baseline, there were substantial associations between anaerobic species, *Corynebacterium* spp., *Staphylococcus* spp., and *Dolosigranulum* spp. Then, a high bioburden reduction was observed after the application of povidone-iodine (log_10_ 3.68 ± 0.69 at 5 min; log_10_ 3.57 ± 0.94 at 2 h; log_10_ 1.17 ± 1.40 at 24 h), compared to the control. The top species affected by the treatment were *Cutibacterium acnes, Staphylococcus,* and *Corynebacterium* species. None of the subjects experienced any adverse effects, nor increases in mucociliary clearance time. Antiseptic solutions applied to the anterior nares can transiently and markedly reduce the bioburden of the nose. The registration number for this clinical trial is NCT05617729.

## Introduction

Healthcare-acquired infections (HAIs) affect 1 out of 31 hospitalized patients with increasing mortality and morbidity^1^. Surgical site infections (SSIs) and pneumonia remain among the most frequent HAIs^1,2^. In this regard, considerable efforts have been made by regional and international regulatory agencies in order to reduce HAIs burden during the last decades^2,3^.

Decolonization of skin and nasal microorganisms is a preventive strategy that has been associated with lower SSI rates^4^. The presence of pathogenic microorganisms in the anterior nares has gained substantial attention due to its association with an increased risk for SSIs^5^. The nasal cavity functions as a transition area between skin and airways^6^. *Staphylococcus aureus*, a well-known member of the skin and nose microbiome^6–8^, is also a leading cause of SSIs^9,10^. Recent studies have suggested the profile of the nose microbiome is a determining factor for the susceptibility to *S. aureus* carriage and, for potential development of subsequent SSIs^6,11,12^. There is little known about the effect of nasal decolonization antiseptic agents on the normal microbiome of nares in general, and in particular the time it takes for the normal flora to return to normal.

We hypothesized the nose microbiome (bioburden and microbial diversity) will be affected using an antiseptic agent. Thus, this randomized clinical trial was designed to assess the bioburden and microbial diversity in the anterior nares of individuals. Then we tested the effect of a nasal antiseptic solution on the nose microbiome and mucosal ciliary function.

## Results

**Figure 1:**
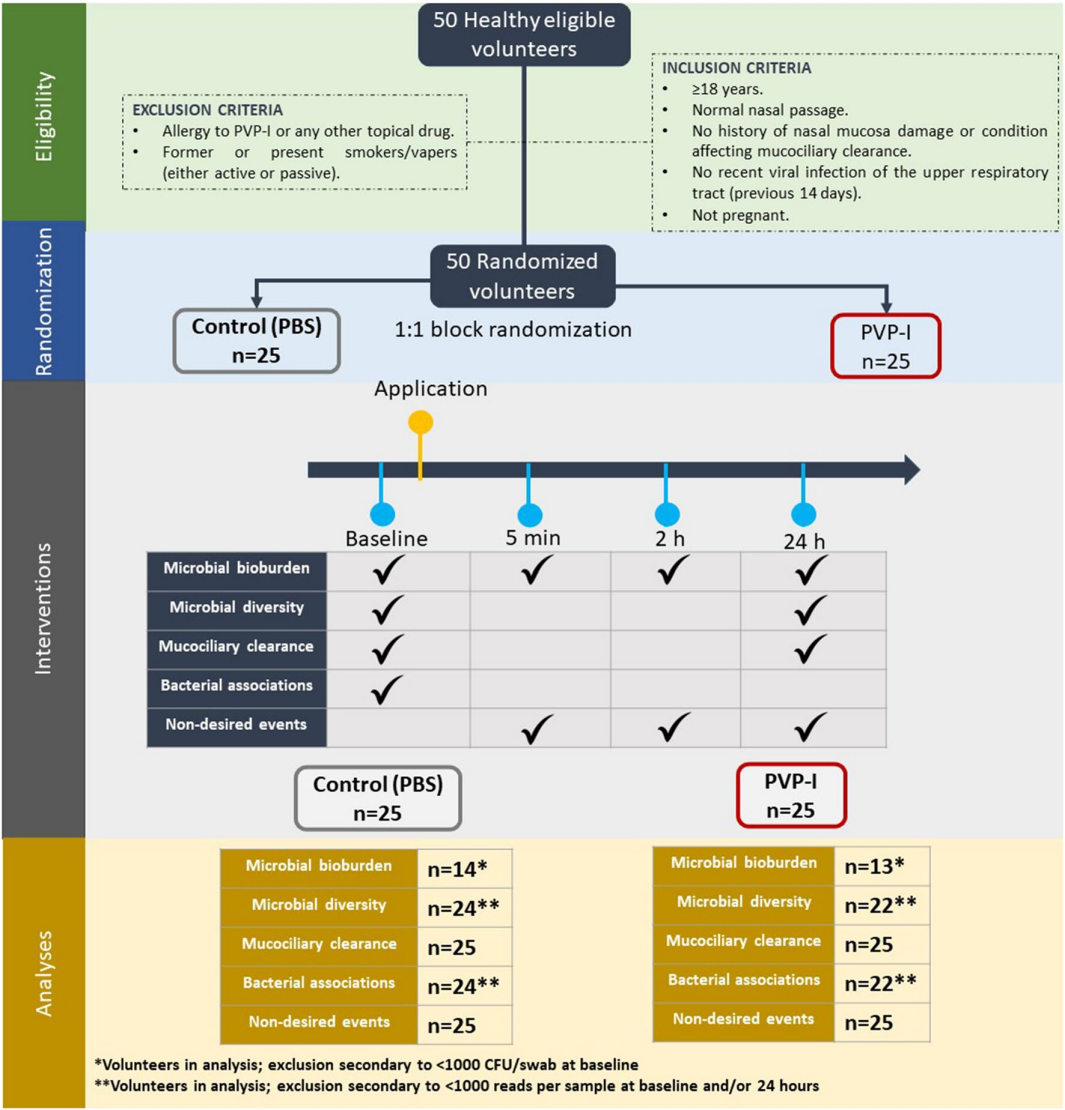
Study design. Fifty volunteers were recruited for this randomized clinical trial. Block randomization was performed to determine group allocation, thus, PVP-I (n = 25) or PBS (n = 25) application in the anterior nares. We assessed bioburden at baseline, 5 min, 2 h, and 24 h post-application, meanwhile microbial diversity and mucociliary clearance was assessed at baseline and 24 h post-application. Bacterial associations were assessed at baseline and non-desired events were examined at every follow-up visit. *Abbreviations: PBS; phosphate-buffered saline, PVP-I; povidone-iodine*.

A total of 50 volunteer subjects were enrolled in the study in March 2022, of whom 19 were females (38%) and their median population age was 26 years (IQR, 24–37). Each group (PVP-I & Control [PBS]) was comprised by 25 participants. We did not exclude any subject; however, samples were excluded according to different criteria described thoroughly in the Methods section (Fig. 1). Moreover, all participants received the intended treatment and completed the interventions throughout this study. As described before, the follow-up visits were scheduled at 2 h and 24 h after application.

### Bioburden reduction

**Figure 2:**
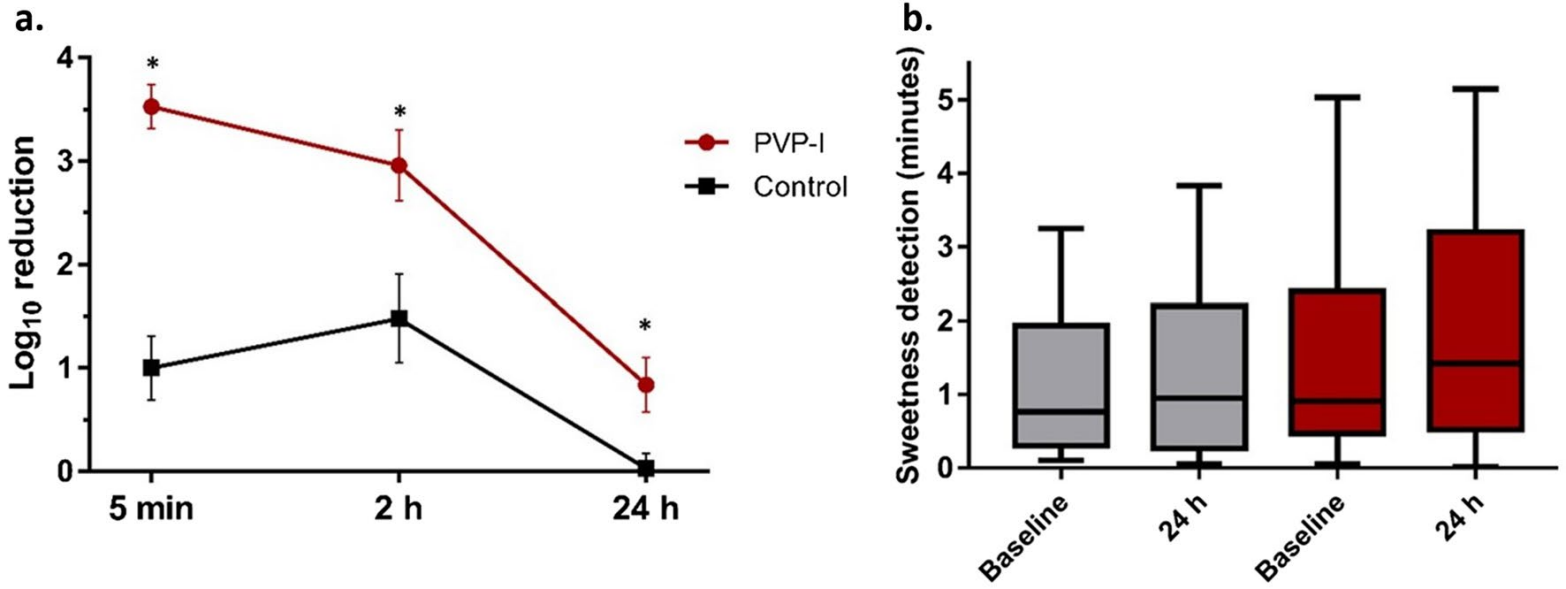
Bioburden reduction (**a**) and mucociliary clearance assessment (**b**), at different timepoints after application of the testing solutions (PVP-I or PBS). (**a**) Data is expressed as mean ± SEM CFU log_10_ reduction, from baseline bioburden, and compared using the Student *t* test. (**b**) Sweetness detection, in minutes, at baseline and 24 h post-application. This plot was performed using R (version 3.0.1, http://www.r-project.org), by the RTL Genomics facility (Lubbock, Texas). Abbreviations: PVP-I; povidone-iodine; *p < *0.05*.

The baseline nose bioburden varied considerably among individuals (3517.5 [IQR, 398.5–13,176] CFU/ml). At 5 min after application of the nasal antiseptic solution, we observed a higher and almost a complete elimination of bacteria from the anterior nares, compared to PBS (log_10_ reduction after PVP-I 3.68 ± 0.69 ***vs****.* log_10_ reduction after PBS 0.94 ± 1.12; *t* statistic, *p* < 0.01; Fig. 2a). At 2 h after the application of PVP-I, bacterial reduction of log_10_ 3.57 ± 0.94 (*t* statistic, *p* < 0.01) was still observed.

Moreover, by 24 h, the reduction in bacterial load was still detected in individuals receiving PVP-I (log_10_ reduction after PVP-I 1.17 ± 1.40 vs. log_10_ reduction after PBS 0.33 ± 1.44;* t* statistic, *p* = 0.14). The bacterial load analysis by 16S qPCR supported the findings from culture, suggesting a major difference between baseline and 24 h post-application, especially after the use of the PVP-I formulation. The mean reduction after 24 h was 0.67 in the PVP-I group (Tukey test, mean difference from baseline − 0.67 [95% CI − 1.21, − 0.14], adjusted *p* = 0.01) and 0.25 after applying PBS (Tukey test, mean difference from baseline − 0.25 [95% CI − 0.77, 0.26], adjusted *p* = 0.56).

### Bacterial diversity throughout timepoints

At baseline, a similar operational taxonomic units (OTUs) *per* sample (Tukey test, − 10.97 [95% CI − 36.96, 15.01], adjusted *p* = 0.67) and Hill1 diversity (Tukey test, − 3.3 [95% CI − 11.61, 5], adjusted *p* = 0.71) was detected between groups. After 24 h, the Hill1 diversity increased in both groups; however, this trend was more noticeable for individuals receiving PVP-I (Tukey test, mean difference from baseline 7.74 [95% CI − 0.74, 16.22], adjusted *p* = 0.08) than those receiving PBS (Tukey test, mean difference from baseline 4.76 [95% CI − 3.36, 12.87], adjusted *p* = 0.41).

**Figure 3:**
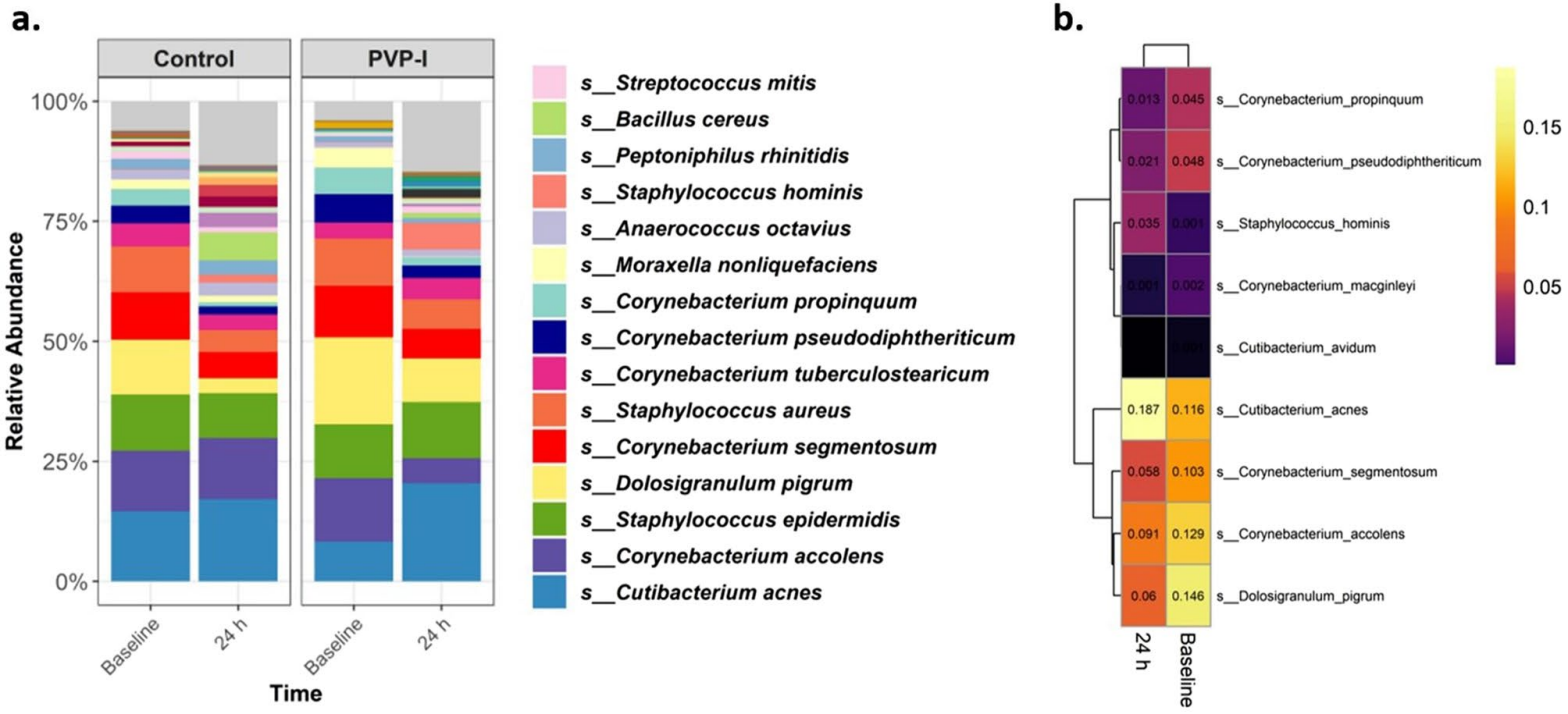
Relative abundance of the most prevalent bacterial species (**a**) and heatmap (**b**) illustrating bacteria detected to be differentially abundant between timepoints by ANCOMBC procedure. A cell colored in black can be considered “true” zero, whereas there were no detections of a particular bacteria for that given cell. This plot was performed using R (version 3.0.1, http://www.r-project.org), by the RTL Genomics facility (Lubbock, Texas). *Abbreviations: PVP-I; povidone-iodine*.

We analyzed the mean relative abundance of the most prevalent bacteria in the entire dataset. Almost 75% of the bacteria detected were *Staphylococcus, Cutibacterium,* and *Corynebacterium* species (Fig. 3a). The weighted Unifrac Principal component analysis (PCA) on bacterial profile did not differ between groups at baseline (pairwiseAdonis based on weighted UniFrac, F = 0.88, R^2^ = 0.02, adjusted *p* = 0.77), neither the unweighted UniFrac PCA (pairwiseAdonis based on unweighted UniFrac, F = 1.57, R^2^ = 0.03, adjusted *p* = 0.21). Moreover, *S. aureus* was found in 11 volunteers at baseline (22%), with no significant difference between groups (5 [20%] in PVP-I group vs. 6 [24%] in PBS group; X^2^, *p* = 0.73).

Overall, the ANCOM-BC procedure identified 9 species varied at 24 h post-application, compared to baseline, including *Cutibacterium acnes, Staphylococcus hominis, Corynebacterium accolens,* among others. To note, a decrease of *Corynebacterium* species was observed (e.g., for *C. accolens* beta − 1.29 ± 0.21 at 24 h, W = − 5.91, adjusted *p* < 0.001), meanwhile there was a rebound in the abundance of *C. acnes* (beta 0.39 ± 0.11 at 24 h, W = 3.37, adjusted *p* = 0.022) and *S. hominis* (beta 1.005 ± 0.20 at 24 h, W = 4.81, adjusted *p* < 0.001; Fig. 3b). Only *C. accolens’* mean relative abundance was identified to decrease after using PVP-I (W = 24.28, adjusted *p* < 0.001), compared to PBS findings.

### Bacterial associations at baseline

**Figure 4:**
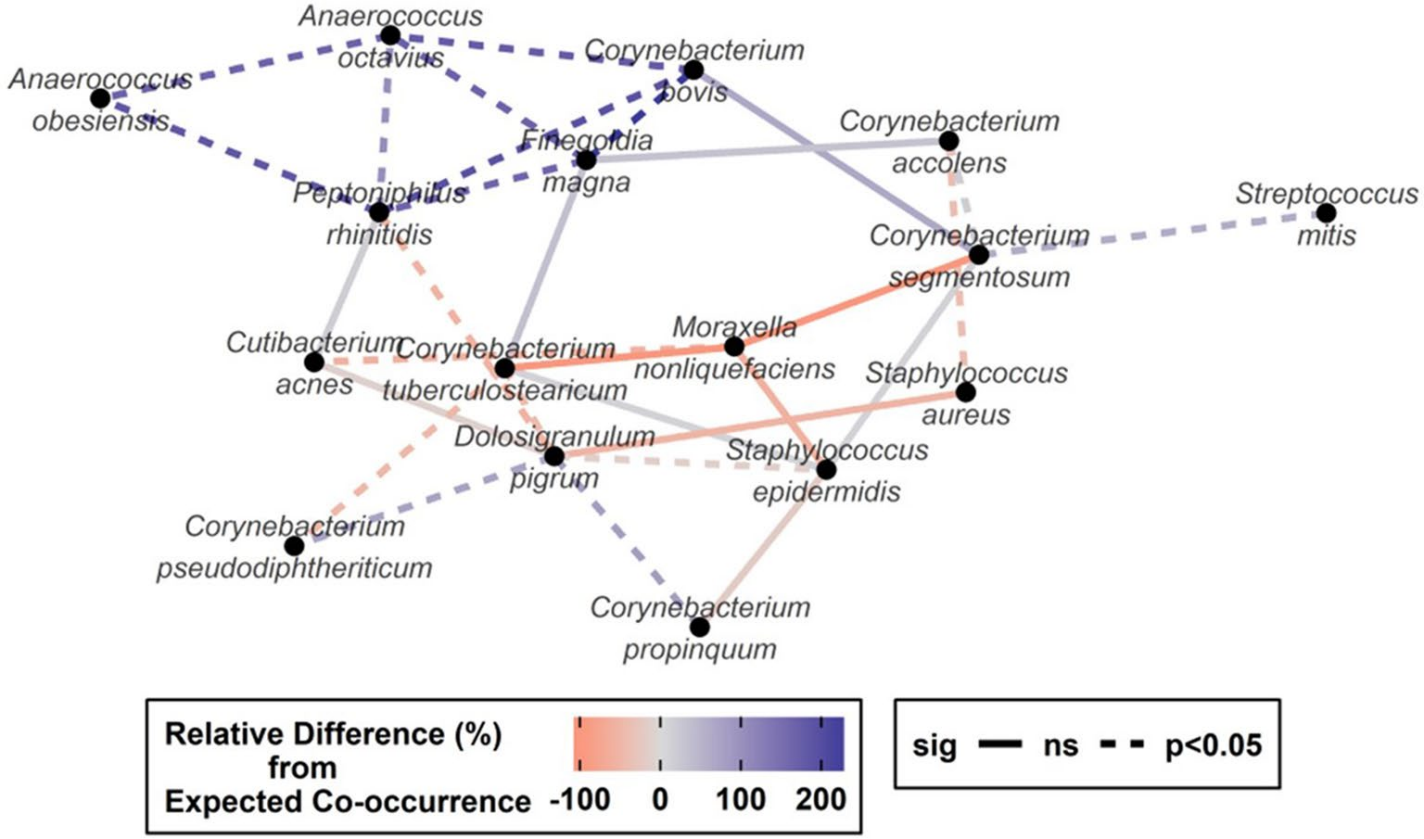
Network plot shows associations among bacterial species with *p* < 0.10. Each node represents a species and is linked by species associations, calculated with Spearman’s rank correlation coefficient. This plot was performed using R (version 3.0.1, http://www.r-project.org), by the RTL Genomics facility (Lubbock, Texas).

A total of 18 paired species associations were marked as being found in co-occurrence at a rate different from expected by random chance (Spearman ranked correlations, *p* < 0.05) whereas an additional 12 species were approaching significance (Spearman ranked correlations, *p* < 0.1; Fig. 4 and Table 1). From the binary perspective, the greatest positive associations were observed among a set of primarily anaerobic species, including *Finegoldia magna, Anaerococcus*, and *Corynebacterium* species. Moreover, *C. accolens* and *Dolosigranulum pigrum* were negatively associated with *S. aureus* (Spearman ranked correlations, r = − 0.151, *p* = 0.031) and *Staphylococcus epidermidis* (Spearman ranked correlations, r = − 0.387, *p* = 0.027) presence, respectively.

**Table 1:** Top 20 Co-occurrence associations at baseline.

sp1	sp2	sp1_inc	sp2_inc	obs_cooccur	exp_cooccur	diff	*p*
Finegoldia_magna	Anaerococcus_octavius	11	17	11	4.1	168.3	< 0.00001
Finegoldia_magna	Peptoniphilus_rhinitidis	11	16	10	3.8	163.2	0.00002
Peptoniphilus_rhinitidis	Anaerococcus_octavius	16	17	12	5.9	103.4	0.00016
Dolosigranulum_pigrum	Corynebacterium_tuberculostearicum	21	28	7	12.8	− 45.3	0.00057
Dolosigranulum_pigrum	Corynebacterium_propinquum	21	12	10	5.5	81.8	0.00295
Corynebacterium_bovis	Peptoniphilus_rhinitidis	5	16	5	1.7	194.1	0.00319
Corynebacterium_bovis	Anaerococcus_octavius	5	17	5	1.8	177.8	0.00451
Dolosigranulum_pigrum	Peptoniphilus_rhinitidis	21	16	3	7.3	− 58.9	0.00799
Corynebacterium_bovis	Finegoldia_magna	5	11	4	1.2	233.3	0.00876
Peptoniphilus_rhinitidis	Anaerococcus_obesiensis	16	4	4	1.4	185.7	0.01115
Anaerococcus_octavius	Anaerococcus_obesiensis	17	4	4	1.5	166.7	0.01458
Dolosigranulum_pigrum	Corynebacterium_pseudodiphtheriticum	21	10	8	4.6	73.9	0.01687
Corynebacterium_accolens	Corynebacterium_segmentosum	32	28	23	19.5	17.9	0.02412
Staphylococcus_epidermidis	Dolosigranulum_pigrum	39	21	15	17.8	− 15.7	0.02752
Corynebacterium_tuberculostearicum	Corynebacterium_pseudodiphtheriticum	28	10	3	6.1	− 0.8	0.02998
Staphylococcus_aureus	Corynebacterium_accolens	10	32	4	7	− 42.9	0.03105
Streptococcus_mitis	Corynebacterium_segmentosum	6	28	6	3.7	62.2	0.04022
Moraxella_nonliquefaciens	Cutibacterium_acnes	3	40	1	2.6	− 61.5	0.04084

**Figure 5:**
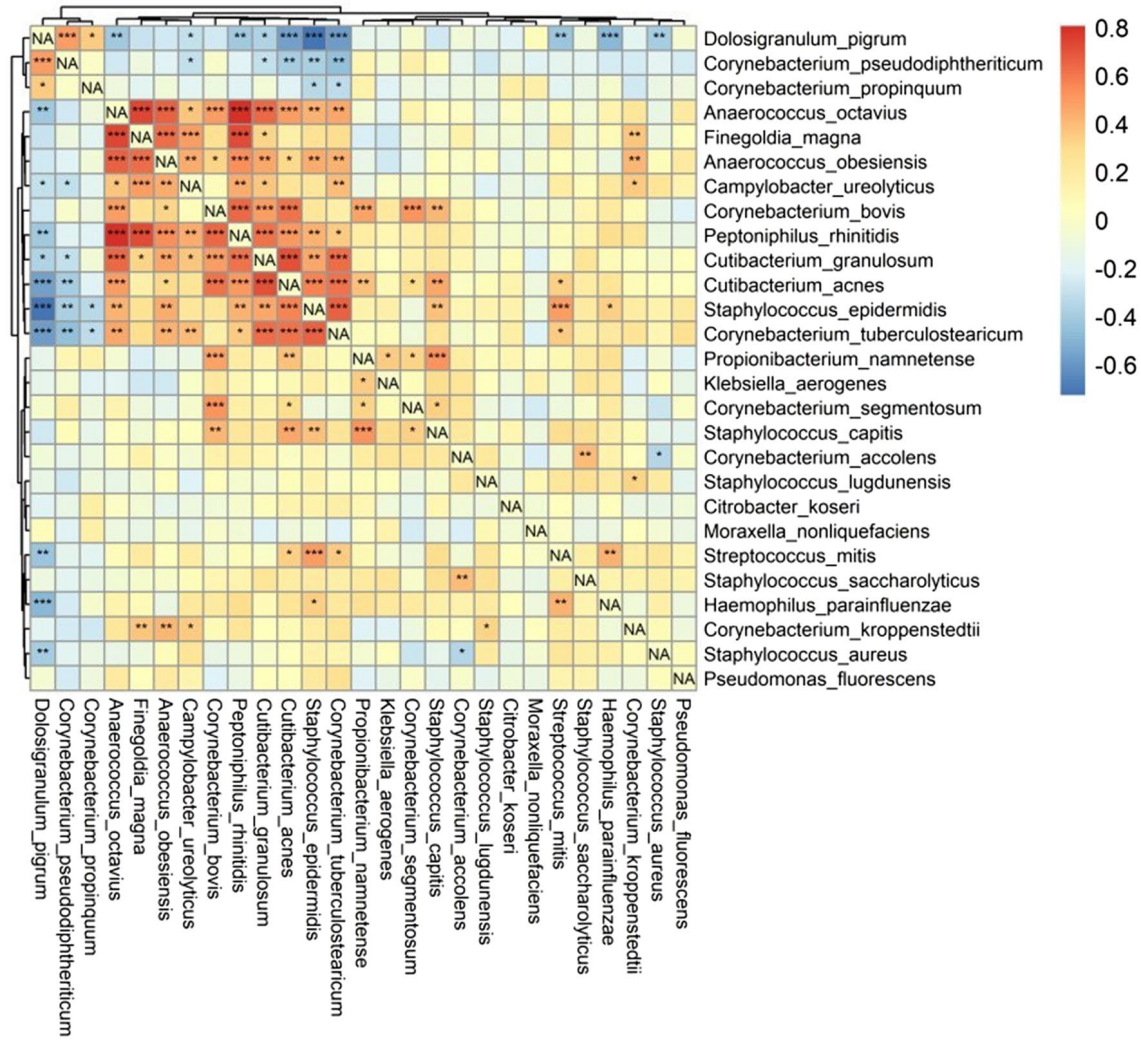
Heatmap shows association between each species observed relative abundance via Spearman correlation coefficient. The Spearman’s correlation coefficient range from − 1 to + 1, whereas values approaching 1 indicate greater strength of association. Significance testing was also performed, whereas asterisks provided per cell indicate the significance threshold achieved per pair tested. Asterisks indicate: * = *p* < 0.05, ** = *p* < 0.01, ***p < 0.001. This plot was performed using R (version 3.0.1, http://www.r-project.org), by the RTL Genomics facility (Lubbock, Texas).

Furthermore, when considering relative abundance, a similar positive trend was observed among anaerobic bacteria (Fig. 5; Spearman ranked correlations, *p* < 0.05). Overall, *Staphylococcus* and *Corynebacterium* species’ abundance showed a positive correlation; however, *S. aureus’* abundance was negatively correlated with both *C. accolens* and *D. pigrum*. *S. epidermidis’* abundance showed the most significant positive correlation with anaerobic species.

### Fungi diversity

Fungi was detected by NGS analysis in half of the samples at baseline. This rate was not modified after the application of PVP-I. The principal fungi detected in our study were *Malassezia* species, with a relative abundance of nearly 80% throughout all samples. The application of PVP-I did not disturb the number of OTUs (Tukey test, mean difference from baseline − 7.0 [95% CI − 21.44, 7.44], adjusted *p* = 0.51) nor Hill1 diversity (Tukey test, mean difference from baseline − 0.19 [95% CI − 3.37, 3], adjusted *p* > 0.99). Moreover, the fungi composition did not vary at the 24-h timepoint, according to the weighted and unweighted UniFrac PCA.

### Mucociliary function and adverse effect assessment

None of the subjects in this study experienced adverse events at any timepoint during the study. Moreover, the mucociliary clearance (MCC) assessment demonstrated that application of PVP-I did not slow the mucociliary function as both groups detected sweetness in a similar timeframe in all time points (Fig. 2b). After 24 h of the nasal PVP-I application, we observed a median of almost a minute and a half to sweetness detection (1.41 min [IQR, 0.52–3.2]), with no statistical difference from the baseline lecture (median 0.90 [IQR, 0.47–2.4] min vs. 1.41 [IQR, 0.52–3.2] min; Mann–Whitney U, *p* = 0.78). A similar trend was appreciated in the PBS group (median 0.76 [IQR, 0.3–1.93] min vs. 0.95 min [IQR, 0.26–2.2]; Mann–Whitney U, *p* = 0.63).

## Discussion

It is well known that nasal and extra-nasal surfaces harbor microorganisms that can cause SSIs^5,6,13^. Thus, nasal decolonization has been shown to be an effective strategy to reduce the bioburden in the nares and impact the rate of SSIs^5,14,15^. Surprisingly, there is little data on the safety and effect of nasal decolonization on the nose bioburden^5,16,17^ and microbial composition^18,19^.

The current study provides a great insight into the nasal microbiome and the effect that a novel povidone-iodine (PVP-I) antiseptic solution had on the bioburden of the nares. An independent formulation was used to avoid confounding related to excipients in commercially available products. Moreover, testing the active ingredient in an independent formulation may enhance the embracement of our results by healthcare systems and the research community.

Using next generation sequencing (NGS), we were able to map out the microbiome composition of the nares. To note, our study targeted the hypervariable regions V1–V2. This is important, since previous studies have demonstrated that the targeted sequence strongly influenced the microbial communities captured by NGS^20–22^, and should be selected based on the sample being studied (*e.g.*, skin, nose, synovial fluid, stool, among others)^20,21,23,24^. The V1–V3 region has shown the highest ability to discriminate *Staphylococcus* species^20,22^ and a fairly similar performance has been observed with the V1–V2 region^21^, with a shorter sequence length. Moreover, it is plausible that these primers gave us a lower discrimination power for *Pseudomonas* and *Neisseria* species^22^, which should be taken in consideration in further comparisons.

The anterior nares of individuals in our study were mainly comprised of *Staphylococcus, Cutibacterium,* and *Corynebacterium* species. The nose mucosa, a transition area between dry and moist areas^6^, has shown a similar composition to sebaceous and moist skin areas, with a remarkable presence of species belonging to these 3 geni^7^. Nearly 25% of individuals in our study harbored *S. aureus* in the anterior nares^11,12^.

We were also interested in studying the inter-organism interaction. Using bioinformatics, we detected that presence of *Corynebacterium* spp., was negatively associated with *S. aureus* colonization, perhaps indicating an inter-species competition^25,26^. In addition, *Dolosigranulum* spp. and the *Gammaproteobacteria* taxon were identified as informative predictors for *S. aureus* non-carriage^12,25,27^. *Dolosigranulum* spp can also affect coagulase-negative *Staphylococcus* species, as demonstrated in our study. Notably, we detected *S. aureus* abundance was negatively correlated with both *C. accolens* and *D. pigrum* abundance*.* Still, the specific mechanisms facilitating or interfering with the microbial interactions in a nutrient-limited mucosa, remain an area of interest to scientists.

The international community has also focused research on microbial interactions within the nose microbiome^12,25,26,28,29^. A recent review thoroughly addressed the nutritional interactions between species of the nose microbiome, pointing out that anaerobic microniches are favored by crypts and the nearby presence of aerobe species, which ultimately deplete oxygen^28,30^. In this regard, our study detected the strongest binary positive associations between *Corynebacterium* spp and a set of anaerobic species at baseline. Moreover, when considering relative abundance, *Corynebacterium* and coagulase-negative *Staphylococcus* species, mainly *S. hominis*, were also positively correlated. Both facts, later supported the positive correlation observed between the abundance of *S. epidermidis* and anaerobic species in our study.

The nose microbiome composition barely relies on host genetics^25^, bringing up the possibility of manipulation by environmental factors. In our study, species belonging to the most abundant genus at baseline were found to vary post-application, even with PBS. The mechanical force of the swab, plus the diluent effect of PBS could explain this latter fact. To note, a detachment of microorganisms was observed in the PBS group (mean log_10_ reduction of 0.94 at 5 min). After a single application of PVP-I, our findings suggested the reduction of pathogenic (*S. aureus*) and easily available bacteria (*Corynebacterium* spp.), and enhancement of more indolent species (anaerobic and coagulase-negative *Staphylococcus*).

Our study is the first to elucidate the effect of a PVP-I based formulation on the nose microbiome. Previous studies have examined the effect of different antimicrobials on nose microbiome^18,19^. After a dual application for 5 days of a mupirocin ointment, Roghmann et al. also found a reduction in the presence and abundance of *S. aureus*. Similar to our results, a regrowth of coagulase-negative *Staphylococcus* species was observed. Nevertheless, the increasing resistance rates associated with this compound pose a major drawback to the extensive use of mupirocin^17,31^. In our study we noted that a single application of PVP-I solution resulted in a significant bioburden reduction even after 5 min and part of this effect lasted as long as 24 h. The effect of PVP-I appeared to be organism specific also. For example, the relative frequency of *C. accolens* decreased while the relative and absolute frequency of *S. epidermidis* was less affected. *C. accolens* depicts a controversial role, since previous authors have pointed out a protective and cooperative role in *S. aureus* carriage^12,26,32,33^. It is also interesting to note that regrowth was mainly attributed to *S. hominis* and *C. acnes* and the application of PVP-I did not disturb the fungal composition in the anterior nares.

Our study also evaluated the effect of PVP-I on mucociliary function and noted that MCC was not at all disrupted. This is important as nasal mucosa plays a critical defense role against pathogens^34,35^. Paralysis of ciliary function of nasal mucosa by an antiseptic agent would be problematic.

Our study has some limitations. First, the culture conditions prioritized aerobic bacteria over strictly anaerobic bacterial and fungal species. Most of these microorganisms are fastidious and difficult to grow even under appropriate culture conditions^36^. This may have required more swabs per timepoint, thus repeated mechanical rubbing, and more complex sampling techniques. We did, however, use targeted metagenomics that should have detected most species regardless of their requirements. In fact, the bacterial load detected by 16S qPCR confirmed our culture findings. Second, our study assessed the effect of a PVP-I-based antiseptic solution exclusively on the anterior nares. This should be taken into consideration as *S. aureus* has also been found in higher and more moist areas^11,12^, where it can interact with other pathogenic microorganisms (e.g., *Pseudomonas aeruginosa* in chronic rhinosinusitis^37^). The reason for limiting the application to anterior nares was to simulate the clinical situation when decolonization by any agents is limited to this area. Also, our study population was relatively young and the microbiome profile of our volunteers may be different to the older patients with comorbidities that require hospitalization^37^. Our study population did not report a history of viral infection within 14 days prior to the start of the trial. However, disruption of the nose microbiome may still be likely if a viral infection happened even before 4 weeks^38,39^, time at which the host´s immune response is also thought to recover to its baseline parameters^38^. In this regard, our results should be taken with caution as our understanding of the nose microbiome interactions with the host and external agents^40^, evolve. Moreover, our sample size was mainly determined by resource constraints rather than a formal sample size estimation. There is a possibility that our study is not adequately powered to study all the outcomes described. For example, a post hoc estimation demonstrated that our study´s power was superior to 80% for the total CFUs reduction at 5 min and 2 h, but fell to 33.6% for the data at 24 h. For this latter timepoint, the bacterial load data by 16S rRNA qPCR may be more reliable. Finally, we only tested the effect of a single intranasal application of the PVP-I antiseptic and results may vary with its repetitive use and/or additional preventive interventions.

## Conclusions

Based on the findings of the current study, it appears that a single application of a PVP-I antiseptic to anterior nares is effective in reducing the bioburden of the nose and eliminating pathogens. This effect is achieved in the presence of normal mucociliary function and without disruption of the microbiome.

## Methods

### Study design

In order to test our hypothesis, we developed an antiseptic formulation containing povidone-iodine (PVP-I) at 10% (w/w) as the active ingredient. Then, we conducted a randomized, placebo-controlled, and parallel-group study in March 2022 (Fig. 1).

This was an unblinded study due to the discrepancy in coloration of the tested formulations with PVP-I (reddish-brown) and the phosphate-buffered saline (PBS; transparent). Nevertheless, the statistical analysis was performed by an independent, blinded statistician. The volunteers were randomized to receive PVP-I (treatment) or PBS (control) in a 1:1 ratio by block randomization. EC generated the random allocation sequence and enrolled the volunteers in this study. DFR & JC assigned and verified the interventions in our trial according to the allocation group. Sequentially numbered badges were used to identify volunteers. DFR & JC matched the badges with the allocation group before and after every intervention. The allocation group was shared to DFR & JC 5 min before the beginning of the trial. All volunteers were followed-up for up to 24 h after the application of the nasal formulation.

All methods were carried out in accordance with Title 21 of the Code of Federal Regulations (CFR), Parts 50 and 56, the Declaration of Helsinki, and Health Insurance Portability and Accountability Act (HIPAA) regulations. Western Institutional Review Board reviewed and approved this clinical study. Verbal and written informed consent were obtained from all subjects participating in this trial. Our study was also registered on the ClinicalTrials.gov platform (NCT05617729, 15/11/2022). This study was reported according to the Consolidated Standards of Reporting Trials (CONSORT) 2010 guidelines.

### Participants

This population-based study comprising healthy volunteers 18 years and older was conducted at Philadelphia, Pennsylvania, USA. A total of 50 volunteer subjects were enrolled in the study. Secondary to a lack of studies assessing the longitudinal changes in microbial diversity after PVP-I application at the planification and conducting stage in our study, a formal sample size calculation was not performed. Instead, considering internal budget and the desired number of timepoints, this study was designed to include a total of 50 volunteers (25 interventional [PVP-I] & 25 control [PBS]).

Eligible participants had a normal nasal passage, did not report any history of nasal mucosa damage (*e.g.*, allergic rhinitis, chronic rhinosinusitis, atrophic rhinitis) or other conditions affecting mucociliary clearance ([MCC]; pain killers, antihistamines, asthma, cystic fibrosis), did not experience a recent viral infection of the upper respiratory tract (previous 14 days), and were not pregnant. Subjects known to be allergic to PVP-I or any other topical drug and former or present smokers/vapers (either active or passive) were also excluded from our clinical trial.

All volunteers signed a consent form and agreed to refrain from blowing and scrubbing the test area with soap, powders, lotions and/or personal care (either cosmetic or toiletry) products for 24 h after the start of this study. All follow-up visits were completed in all volunteers.

### Interventions

All the interventions described in this study were performed using the same batch of antiseptic formulation containing PVP-I (#021,622-01). The clinical comparison was conducted using a negative control solution, namely PBS.

The volunteers were enrolled to calculate the bioburden at baseline, 5 min, 2 h, and 24 h post-application. Meanwhile, microbial diversity and MCC were assessed at baseline and 24 h post-application. Figure 1, summarizes all the interventions throughout the study. In addition, we collected demographic data and examined the volunteers for non-desired effects at every follow-up visit.

The swabbing and application area covered the anterior nares, the first 2–3 cm inside the nose. It consisted of a rotatory movement of the cotton tip of the swab throughout the nasal mucosa for ~ 15 s per nostril. Baseline and post-application samples were collected using Flocked FLOQSwabs (COPAN Diagnostics Inc, CA, US). After the baseline measurement, the testing solutions were applied with a standard cotton swab with polystyrene handle (Puritan, ME, USA). According to allocation, an approximate volume of 0.25 mL of the testing solution was applied per nostril. Sterile gloves were used at all times and changed between timepoints, after applying the test product (PVP-I or PBS), and between volunteers.

The flocked swabs were sealed and immediately processed to determine bioburden. With an aseptic technique, swabs were immersed in 1 mL of sterile PBS and streaked onto Trypticase Soy Agar plates. Then, swabs were stored at 4 °C until the study completion. All plates were incubated at 37 °C under aerobic conditions for 48 h. Growth was assessed by counting the colony-forming units (CFUs) at 48 h of incubation.

Likewise, nasal swabs (FLOQSwabs; COPAN Diagnostics Inc, CA, US) were sealed and sent overnight to perform NGS analysis at the MicrogenDx facility (Lubbock, Texas), similar to previous studies^41,42^. Samples were mechanically lysed using the Qiagen TissueLyser (Qiagen, Hilden, Germany). Then, each sample was spiked with a positive internal control to ensure the success of the extraction process. In addition, negative controls were run along with samples to identify contamination associated with the extraction process. DNA was then extracted using a KingFisher Flex Purification System (ThermoFisher Scientific, Waltham, MA, US). DNA was then amplified using the 28F (GAGTTTGATCNTGGCTCAG) and 388R (GCTGCCTCCCGTAGGAGT) primers specific to the hypervariable regions V1–V2 in the 16S rRNA gene for bacteria. The Internal transcribed spacer (ITS) gene for fungi was assessed for ITS3-4 using ITS3F (GCATCGATGAAGAACGCAGC) and ITS4R (GCATCGATGAAGAACGCAGC) primers. The amplicons were created on a LightCycler 480 II (Roche Life Sciences, Indianapolis, IN, US) with the following thermal cycling profile: 95 °C for 5 min; 35 cycles of 94 °C for 30 s, 52 °C for 40 s, and 72 °C for 1 min; and final extension at 72 °C for 10 min. Amplified DNA was then pooled and run on the Illumina MiSeq (Illumina Inc, San Diego, CA) at 2 × 250 bp.

We also examined MCC using the saccharin test^43,44^. A cotton swab with a wooden handle (Puritan, ME, USA) was immersed in saccharin powder (Spectrum, NJ, USA) and carefully introduced below the anterior end of the inferior turbinate. We recorded the time (minutes) between the placement of the saccharin and the volunteer´s perception of sweetness. Baseline and/or 24-h times above 30 min were considered abnormal.

### Main outcomes and measures

The primary outcomes assessed were bioburden, microbial diversity and MCC (Fig. 1). Total CFU counts were determined at baseline, 5 min, 2 h, and 24 h post-application. No distinction was made between colony morphologies. CFUs were converted to log_10_ and bioburden change was calculated from the baseline measure.

On the other hand, microbial diversity and MCC were assessed at baseline and 24 h post-application. Alpha*-*diversity, which refers to the number of species or species richness detected, and beta-diversity, which refers to the number of unique species, parameters were calculated and compared between groups and timepoints. For MCC, the time to sweetness detection was reported in minutes.

In addition, as secondary outcomes, we assessed the bacterial species’ relative and absolute frequency at baseline, in order to determine microbial associations, and volunteers were examined for any non-desired effects at every follow-up visit (5 min, 2 h, and 24 h post-application).

### General statistical analysis

Categorical variables were summarized as absolute and relative frequencies. Comparisons were performed using the X^2^ test. Quantitative data was assessed for normality with the Shapiro–Wilk test. Mean ± standard error of the mean or median (interquartile range [IQR]) were reported, accordingly. Total CFU counts were independently determined by 2 experienced members of the laboratory personnel with an inter and intra-reviewer Cohen´s kappa statistic score > 0.80. Volunteers with 1 × 10^3^ or more CFUs at baseline were included in the bioburden change calculation. Comparisons were performed using the Student´s t-test. A two-tailed *p*-value < 0.05 was considered statistically significant.

### Next-generation sequencing analysis

All analyses were conducted in R (R Core Team, http://www.r-project.org, Vienna, Austria), using the vegan^45^ and phyloseq^46^ packages. The operational taxonomic units (OTUs) were determined using the UPARSE algorithm^47^ against the MicroGenDX high- internal database, which was adapted from the National Center for Biotechnological Information (NCBI), with additional curation. During pre-processing, OTUs described as ‘No hit’ or *Ralstonia* (a common reagent contaminant) study-wide were removed prior to analysis.

The initial read count was normalized to 1000 reads per sample using Scaling with Ranked Subsampling (SRS). All samples that fell below the 1000 read cutoff were dropped and only samples with completeness through the 2 time points were kept (92 out of 100). Rarefaction and quality control were performed with the intent to assure the quality of data being analyzed and minimize false discoveries. To ensure the transparency of this process, a summary of statistics before and after count normalization can be found in the Supplementary file.

Alpha diversity was examined from three perspectives: overall richness (number of OTUs), Shannon (H′) diversity, and the Hill number of effective species. Measures of alpha diversity were screened for group differences using an analysis of variance (ANOVA). Pending significant effects from the global ANOVA, pairwise post hoc testing was performed using Tukey’s Honest Significant Difference (HSD) test, unless width restrictions were detected.

UniFrac distances were calculated using the phyloseq package in R^46^.

Multivariate differences among locations were evaluated with “Permutational Multivariate Analysis of Variance Using Distance Matrices,” function *adonis*^45^. For ADONIS, distances among samples first were calculated using unweighted (presence and absence of OTUS) or weighted (abundance of OTUs) UniFrac, and then an ANOVA-like simulation was conducted to test for location differences. Pairwise multivariate differences among groups were evaluated with function “pairwise.adonis”^48^ upon detection of significant factors with ADONIS. False Discovery Rate (FDR) p-value adjustment was used to correct for multiple testing.

Arguments for global ANCOM-BC^49^ for the species and genus level included zero cut = 0.80, struc zero = F, and neg lb = F. In this way, taxa with less than 20% prevalence were filtered out from ANCOM-BC analysis. Results for all pairwise tests were collected and a new *p*-value adjustment using Holm’s method. As previously described^49^, the criteria for calling taxa differentially abundant was based on the adjusted *p*-value < 0.05.

Finally, we performed a co-ocurrence analysis, to determine whether any bacterial species were significantly associated with *S. aureus* carriage. The co-occurrence analysis was performed using the R package cooccur, from a binary (presence/absence) perspective. To reduce likelihood of spurious associations, species were filtered to include only those found in at least 5% of remaining samples and above 1% relative abundance per sample. After all filtering, 27 species across 46 samples remained for analysis. To investigate from a perspective incorporating the observed relative abundance of each species, a correlation analysis was performed where each species was screened against others via Spearman ranked correlations.

Figures 2, 3, 4 and 5 were created in R software (version 3.0.1, http://www.r-project.org), by the RTL Genomics (Lubbock, Texas) facility.

### Supplementary Information

Supplementary Information.

## Data Availability

All sequencing data from this study is available through the National Center for Biotechnological Information (NCBI) BioProject and Sequence Read Archive (SRA) databases under accession number PRJNA1024520. The data can also be accessed through the following direct link https://dataview.ncbi.nlm.nih.gov/object/PRJNA1024520?reviewer=naeng581gqldr80pi2rkssag5p. The protocol and datasets used and/or analyzed during the current study are available from the corresponding author on reasonable request.

## Code availability

The underlying code for this study is not publicly available but may be made available to qualified researchers on reasonable request from the corresponding author, and with permission of MicrogenDx (Lubbock, Texas) and Research and Testing Laboratories (RTL), LLC (Lubbock, Texas).

## References

[1] Magill, S. S. *et al.* Changes in prevalence of health care-associated infections in U.S. Hospitals. *N. Engl. J. Med.* **379**, 1732–1744 (2018). 30380384 10.1056/NEJMoa1801550 PMC7978499

[2] CDC. *HAI Data*. (2019). at https://www.cdc.gov/hai/data/index.html

[3] World Health Organization. in (eds Ducel, G., Fabry, J. & Nicolle, L.) (World Health Organization, 2002). https://apps.who.int/iris/handle/10665/67350

[4] Reagan, K. A. *et al.* You get back what you give: Decreased hospital infections with improvement in CHG bathing, a mathematical modeling and cost analysis. *Am. J. Infect. Control* **47**, 1471–1473 (2019). 31400883 10.1016/j.ajic.2019.07.003

[5] Bode, L. G. M. *et al.* Preventing surgical-site infections in nasal carriers of *Staphylococcus aureus*. *N. Engl. J. Med.* **362**, 9–17 (2010). 20054045 10.1056/NEJMoa0808939

[6] Krismer, B., Weidenmaier, C., Zipperer, A. & Peschel, A. The commensal lifestyle of *Staphylococcus aureus* and its interactions with the nasal microbiota. *Nat. Rev. Microbiol.* **15**, 675–687 (2017). 29021598 10.1038/nrmicro.2017.104

[7] Grice, E. A. *et al.* Topographical and temporal diversity of the human skin microbiome. *Science* **324**, 1190–1192 (2009). 19478181 10.1126/science.1171700 PMC2805064

[8] Moss, B. & Squire, J. R. Nose and skin carriage of Staphylococcus aureus in patients receiving penicillin. *Lancet (London, England)* **1**, 320–325 (1948). 18905395 10.1016/S0140-6736(48)92088-1

[9] Otter, J. A. & French, G. L. Community-associated meticillin-resistant *Staphylococcus aureus* strains as a cause of healthcare-associated infection. *J. Hosp. Infect.* **79**, 189–193 (2011). 21741111 10.1016/j.jhin.2011.04.028

[10] Weiner, L. M. *et al.* Antimicrobial-resistant pathogens associated with healthcare-associated infections: Summary of data reported to the national healthcare safety network at the Centers for disease control and prevention, 2011–2014. *Infect. Control Hosp. Epidemiol.* **37**, 1288–1301 (2016). 27573805 10.1017/ice.2016.174 PMC6857725

[11] Kaspar, U. *et al.* The culturome of the human nose habitats reveals individual bacterial fingerprint patterns. *Environ. Microbiol.* **18**, 2130–2142 (2016). 25923378 10.1111/1462-2920.12891

[12] Yan, M. *et al.* Nasal microenvironments and interspecific interactions influence nasal microbiota complexity and *S. aureus* carriage. *Cell Host Microbe* **14**, 631–640 (2013). 24331461 10.1016/j.chom.2013.11.005 PMC3902146

[13] Grice, E. A. & Segre, J. A. The skin microbiome. *Nat. Rev. Microbiol.* **9**, 244–253 (2011). 21407241 10.1038/nrmicro2537 PMC3535073

[14] Berríos-Torres, S. I. *et al.* Centers for disease control and prevention guideline for the prevention of surgical site infection, 2017. *JAMA Surg.* **152**, 784–791 (2017). 28467526 10.1001/jamasurg.2017.0904

[15] Huang, S. S. *et al.* Chlorhexidine versus routine bathing to prevent multidrug-resistant organisms and all-cause bloodstream infections in general medical and surgical units (ABATE Infection trial): A cluster-randomised trial. *Lancet (London, England)* **393**, 1205–1215 (2019). 30850112 10.1016/S0140-6736(18)32593-5 PMC6650266

[16] Anderson, M. J. *et al.* Efficacy of skin and nasal povidone-iodine preparation against mupirocin-resistant methicillin-resistant *Staphylococcus aureus* and *S. aureus* within the anterior nares. *Antimicrob. Agents Chemother.* **59**, 2765–2773 (2015). 25733504 10.1128/AAC.04624-14 PMC4394816

[17] Baratz, M. D., Hallmark, R., Odum, S. M. & Springer, B. D. Twenty percent of patients may remain colonized with methicillin-resistant staphylococcus aureus despite a decolonization protocol in patients undergoing elective total joint arthroplasty. *Clin. Orthop. Relat. Res.* **473**, 2283–2290 (2015). 25690169 10.1007/s11999-015-4191-3 PMC4457751

[18] Roghmann, M.-C. *et al.* Effect of mupirocin for Staphylococcus aureus decolonization on the microbiome of the nose and throat in community and nursing home dwelling adults. *PLoS One* **16**, e0252004 (2021). 34101737 10.1371/journal.pone.0252004 PMC8186807

[19] McAnally, T. P., Lewis, M. R. & Brown, D. R. Effect of rifampin and bacitracin on nasal carriers of *Staphylococcus aureus*. *Antimicrob. Agents Chemother.* **25**, 422–426 (1984). 6732212 10.1128/AAC.25.4.422 PMC185544

[20] Meisel, J. S. *et al.* Skin microbiome surveys are strongly influenced by experimental design. *J. Invest. Dermatol.* **136**, 947–956 (2016). 26829039 10.1016/j.jid.2016.01.016 PMC4842136

[21] Isabel, A.-S. *et al.* Primer, pipelines, parameters: Issues in 16S rRNA gene sequencing. *mSphere* **6**, 10–1128. 10.1128/msphere.01202-20 (2021). 10.1128/msphere.01202-20 PMC8544895 33627512

[22] Conlan, S., Kong, H. H. & Segre, J. A. Species-level analysis of DNA sequence data from the NIH human microbiome project. *PLoS One* **7**, e47075 (2012). 23071716 10.1371/journal.pone.0047075 PMC3468466

[23] Fernández-Rodríguez, D. *et al.* Human knee has a distinct microbiome: Implications for periprosthetic joint infection. *J. Arthroplasty*10.1016/j.arth.2023.03.084 (2023). 37003456 10.1016/j.arth.2023.03.084

[24] Structure, function and diversity of the healthy human microbiome. *Nature* **486,** 207–214 (2012). 10.1038/nature11234 PMC3564958 22699609

[25] Liu, C. M. *et al.* Staphylococcus aureus and the ecology of the nasal microbiome. *Sci. Adv.* **1**, e1400216 (2022). 10.1126/sciadv.1400216 PMC4640600 26601194

[26] Bassiouni, A. *et al.* Microbiotyping the sinonasal microbiome. *Front. Cell. Infect. Microbiol.* **10**, 137 (2020). 32322561 10.3389/fcimb.2020.00137 PMC7156599

[27] Cole, A. L. *et al.* Identification of nasal gammaproteobacteria with potent activity against *Staphylococcus aureus*: Novel insights into the ‘noncarrier’ state. *mSphere* **6**, 10–1128 (2021). 10.1128/mSphere.01015-20 PMC7802429 33408227

[28] Adolf, L. A. & Heilbronner, S. Nutritional interactions between bacterial species colonising the human nasal cavity: Current knowledge and future prospects. *Metabolites* **12**, 489 (2022). 35736422 10.3390/metabo12060489 PMC9229137

[29] Claudia, L., Andreas, P. & Bernhard, K. *Staphylococcus aureus* colonization of the human nose and interaction with other microbiome members. *Microbiol. Spectr.* **7**, 7.2 (2019). 10.1128/microbiolspec.gpp3-0029-2018 PMC11590430 31004422

[30] Ali, M. Y. Histology of the human nasopharyngeal mucosa. *J. Anat.* **99**, 657–672 (1965). 5857093 PMC1270703

[31] Deshpande, L. M., Fix, A. M., Pfaller, M. A. & Jones, R. N. Emerging elevated mupirocin resistance rates among staphylococcal isolates in the SENTRY Antimicrobial Surveillance Program (2000): Correlations of results from disk diffusion, Etest and reference dilution methods. *Diagn. Microbiol. Infect. Dis.* **42**, 283–290 (2002). 12007448 10.1016/S0732-8893(01)00328-5

[32] Menberu, M. A. *et al.* Corynebacterium accolens has antimicrobial activity against *Staphylococcus aureus* and methicillin-resistant *S. aureus* pathogens isolated from the sinonasal niche of chronic rhinosinusitis patients. *Pathogens (Basel, Switzerland)* **10**, 207 (2021). 33672855 10.3390/pathogens10020207 PMC7918835

[33] Uehara, Y. *et al.* Bacterial interference among nasal inhabitants: eradication of *Staphylococcus aureus* from nasal cavities by artificial implantation of *Corynebacterium* sp.. *J. Hosp. Infect.* **44**, 127–133 (2000). 10662563 10.1053/jhin.1999.0680

[34] Benam, K. H., Vladar, E. K., Janssen, W. J. & Evans, C. M. Mucociliary defense: Emerging cellular, molecular, and animal models. *Ann. Am. Thorac. Soc.* **15**, S210–S215 (2018). 30431350 10.1513/AnnalsATS.201806-439AW PMC6322027

[35] Munkholm, M. & Mortensen, J. Mucociliary clearance: Pathophysiological aspects. *Clin. Physiol. Funct. Imaging* **34**, 171–177 (2014). 24119105 10.1111/cpf.12085

[36] Dunne, W. M. J., Westblade, L. F. & Ford, B. Next-generation and whole-genome sequencing in the diagnostic clinical microbiology laboratory. *Eur. J. Clin. Microbiol. Infect. Dis. Off. Publ. Eur. Soc. Clin. Microbiol.* **31**, 1719–1726 (2012). 10.1007/s10096-012-1641-7 22678348

[37] Dimitri-Pinheiro, S., Soares, R. & Barata, P. The microbiome of the nose-friend or foe?. *Allergy Rhinol. (Providence)* **11**, 2152656720911605 (2020). 32206384 10.1177/2152656720911605 PMC7074508

[38] Wu, Z.-B. *et al.* Dynamic interaction between mucosal immunity and microbiota drives nose and pharynx homeostasis of common carp (*Cyprinus carpio*) after SVCV infection. *Front. Immunol.* **12**, 769775 (2021). 34804060 10.3389/fimmu.2021.769775 PMC8601392

[39] Xu, R. *et al.* Temporal association between human upper respiratory and gut bacterial microbiomes during the course of COVID-19 in adults. *Commun. Biol.* **4**, 240 (2021). 33603076 10.1038/s42003-021-01796-w PMC7893062

[40] Flynn, M. *et al.* Interactions of the bacteriome, virome, and immune system in the nose. *FEMS Microb.* **3**, xtac020 (2022). 10.1093/femsmc/xtac020 PMC10117739 37332500

[41] Goswami, K. *et al.* Comparative meta-omics for identifying pathogens associated with prosthetic joint infection. *Sci. Rep.* **11**, 23749 (2021). 34887434 10.1038/s41598-021-02505-7 PMC8660779

[42] Tipton, C. D. *et al.* Patient genetics is linked to chronic wound microbiome composition and healing. *PLoS Pathog.* **16**, e1008511 (2020). 32555671 10.1371/journal.ppat.1008511 PMC7302439

[43] Andersen, I., Camner, P., Jensen, P. L., Philipson, K. & Proctor, D. F. A comparison of nasal and tracheobronchial clearance. *Arch. Environ. Health* **29**, 290–293 (1974). 4472935 10.1080/00039896.1974.10666589

[44] Rutland, J. & Cole, P. J. Nasal mucociliary clearance and ciliary beat frequency in cystic fibrosis compared with sinusitis and bronchiectasis. *Thorax* **36**, 654–658 (1981). 7314040 10.1136/thx.36.9.654 PMC471692

[45] OHara, R. B., Simpson, G., Solymos, P., Stevens, M. H. H. & Wagner, H. vegan: Community Ecology PackageR package version 1.17–8. (2011).

[46] McMurdie, P. J. & Holmes, S. phyloseq: An R package for reproducible interactive analysis and graphics of microbiome census data. *PLoS One* **8**, e61217 (2013). 23630581 10.1371/journal.pone.0061217 PMC3632530

[47] Edgar, R. C. UPARSE: Highly accurate OTU sequences from microbial amplicon reads. *Nat. Methods* **10**, 996–998 (2013). 23955772 10.1038/nmeth.2604

[48] Martinez Arbizu, P. pairwiseAdonis: Pairwise multilevel comparison using adonis. *R Package version 0.4* (2020).

[49] Lin, H. & Peddada, S. Das. Analysis of compositions of microbiomes with bias correction. *Nat. Commun.* **11**, 3514 (2020). 32665548 10.1038/s41467-020-17041-7 PMC7360769

