# Supplementary material for: Nasal microbiome and the effect of nasal decolonization with a novel povidone-iodine antiseptic solution: a prospective and randomized clinical trial
Source: Sci Rep. 2024 Jul 20;14:16739. doi: 10.1038/s41598-023-46792-8 (PMC11271270; doi:10.1038/s41598-023-46792-8)
Supplement: Supplementary file 1 — Supplementary Information. [file 41598_2023_46792_MOESM1_ESM.pdf]

Nasal Microbiome and the Effect of Nasal Decolonization with a Novel Povidone-Iodine Antiseptic Solution: A Prospective and Randomized Clinical Trial

Diana Fernández-Rodríguez MD, PhD, Jeongeun Cho BS, Emanuele Chisari MD, PhD,

Martin J Citardi MD, Javad Parvizi MD, FRCS

Rarefaction and quality control were performed with the intent to assure the quality of data being analyzed and minimize false discoveries.

For bacteria, initially the read count of the study ranged from 777 to 31, 823. Rarefaction survey was conducted by subsampling OTU richness at variable read depth, from 100 reads through 1,000 reads in stepwise increments of 100 reads. Initial counts were normalized to 1000 reads per sample using Scaling with Ranked Subsampling (SRS).

This procedure was replicated for fungi (initial read count from 112 to 21 411), with a rarefaction survey, from 50 reads through 800 reads in stepwise increments of 50 reads.

A summary table of the Good´s coverage index before and after count normalization can be find below (Table S1). Dropping the samples was based on both, the SRS procedure and their completeness through the 2 time points (baseline and 24 h). The samples dropped had substantially lower coverage and/or read depth, which allowed most communities to remain sampled at above 90% even after normalization.

Table S1. Good´s coverage index before and after count normalization.

	Good´s coverage index	
	Before normalization and quality control	After normalization and quality control
Min.	91.75	86.9
1st Qu.	96.94	91.08
Median	98.1	93.05
Mean	97.77	93.26
3rd Qu.	99.19	95.2
Max.	99.83	98.8

Moreover, a figure showing OTU richness before and after SRS, compared to sequence depth of each sample highlights the utility of this procedure (Figure S1). Before SRS, alpha diversity (OTU richness) was significantly related to sequencing depth, which could have strongly confounded any associations that we would have attempted to make with actual

study variables. As seen in Figure S1, the influence of read depth heterogeneity was sharply reduced following normalization, with the correlation coefficient decreasing from 0.7 to 0.2.

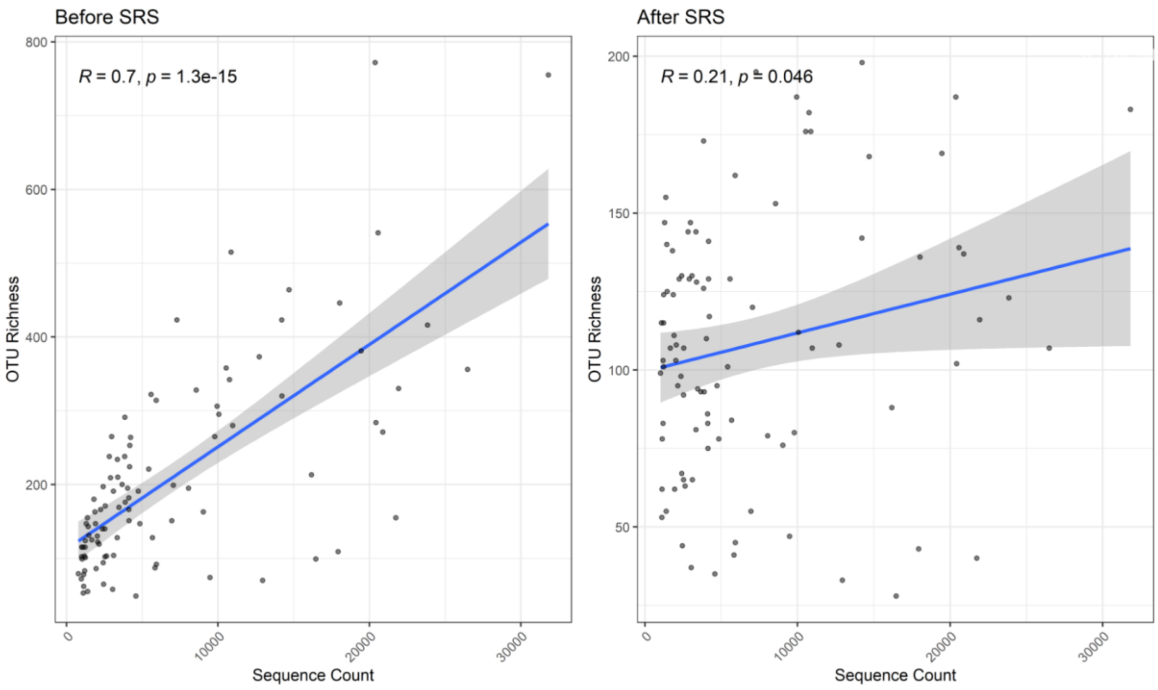

Figure S1. Correlation between OUT richness and sequence count before and after count normalization.
